# How to Get a Grant From the National Institute on Aging

**DOI:** 10.1093/geroni/igaa057.3026

**Published:** 2020-12-16

**Authors:** Kenneth Santora

**Affiliations:** National Institute on Aging, Bethesda, Maryland, United States

## Abstract

Dr. Santora will provide an overview of grant mechanisms available at the National Institute on Aging, with an emphasis on mechanisms of value to early career researchers.

